# Relationship between antihypertensive drug use and number of people with high blood pressure in FY 2018: a descriptive epidemiological study based on the National Database of Health Insurance Claims and Specific Health Checkups of Japan open data

**DOI:** 10.1186/s40780-023-00317-7

**Published:** 2023-12-09

**Authors:** Kanako Mizuno, Ryo Inose, Yukina Yoshimura, Yuichi Muraki

**Affiliations:** Laboratory of Clinical Pharmacoepidemiology, Kyoto Pharmaceutical University, 5, Misasagi-Nakauchicho, Yamashina-ku, Kyoto-shi, Kyoto, 607-8414 Japan

**Keywords:** National Database of Health Insurance Claims and Specific Health Checkups of Japan open data, Antihypertensive drug, Defined daily doses/1000 inhabitants/day, Systolic blood pressure, Diastolic blood pressure, Hypertension

## Abstract

**Background:**

In most countries barring Japan, antihypertensive drug use has been reported using the defined daily doses/1000 inhabitants/day (DID). Although DID has been shown to allow for the assessment of the number of patients treated with a particular drug, the relationship between DID and the number of patients with hypertension has not been clarified. This study aimed to clarify the relationship between antihypertensive drug use and the number of people with high blood pressure based on the National Database of Health Insurance Claims and Specific Health Checkups of Japan (NDB) open data.

**Methods:**

DID was calculated by extracting the use of oral antihypertensive drugs from outpatient prescriptions in the NDB Open Data in FY 2018. The number of people with high blood pressure was calculated using the number of enrollees in each sex–age group for systolic and diastolic blood pressure in the 40–74 years age group. The correlation between the DID of antihypertensive drugs and the number of people with high blood pressure by sex and age class was evaluated using Spearman’s rank correlation coefficient.

**Results:**

The use of antihypertensive drugs increased with age in both men and women. Furthermore, in both sexes, dihydropyridine derivatives, calcium antagonists, and angiotensin II receptor blockers were the main drugs used from the age of 20 years onward. In addition, a very strong positive correlation was found between the number of people with high systolic blood pressure and DID in both sexes (men: *r* = 1, *P* < 0.05; women: *r* = 1, *P* < 0.05). In contrast, there was no significant correlation between the number of people with high diastolic blood pressure and DID in both sexes (men: *r* = − 0.214, *P* > 0.05; women: *r* = 0.393, *P* > 0.05).

**Conclusions:**

To our knowledge, this study is the first to investigate the use of oral antihypertensive drugs in outpatient settings in Japan. In addition, the DID of antihypertensive drugs can be used as an alternative indicator of the number of people with high systolic blood pressure.

## Background

The number of patients with hypertension worldwide has doubled in approximately 30 years as the population grows and ages [1]. Hypertension is a lifestyle-related disease known to cause complications including cerebrovascular diseases and renal dysfunction [2]. Among these, cerebrovascular disease is the fourth leading cause of death in Japan, with hypertension considered its greatest risk factor [3]. Therefore, the treatment of hypertension is extremely important for the prevention of cerebrovascular disease and should be based on guidelines for the management of hypertension. The guidelines for the management of hypertension in Japan published in 2019 recommend the selection of antihypertensive drugs according to the presence of concomitant diseases and specific patient background characteristics, such as elderly age, pregnancy, and age of less than 18 years. Therefore, the antihypertensive drugs used should be identified and monitored.

The World Health Organization (WHO) recommends defined daily doses (DDD)/1000 inhabitants/day (DID) as an indicator for assessing drug use [4]. Therefore, in the 27 member countries of the Organization for Economic Co-operation and Development (OECD), DID values for antihypertensive drug use have been published over time [5]. Antihypertensive drug use varies across countries. In some OECD member countries, calcium (Ca) channel blockers are used more frequently, whereas in others, the use of angiotensin-converting enzyme (ACE) inhibitors or angiotensin II receptor blockers (ARBs) is more frequent. Although Japan is a member of the OECD, the DID for antihypertensive drugs in the country has not been clarified, making comparisons with the rest of the world difficult. In addition, DID has been shown to help assess the number of patients being treated with a particular drug [6]. However, the relationship between the DID of antihypertensive drugs and number of hypertensive patients has not been clarified.

To date, drug use has been evaluated using information such as sales data [7] or the National Database of Health Insurance Claims and Specific Health Checkups of Japan (NDB) [8]. The NDB contains various data including the number of drug prescriptions and laboratory values during specific health checkups. Therefore, in Japan, which has universal health insurance, the NDB data can be used to understand the medical trends in the population at a rate close to that in the total number of general populations [9]. However, NDB users are required to ensure a high-security environment, which is a considerable barrier to the general use of these data [9]. In October 2016, the Ministry of Health, Labour and Welfare (MHLW) published statistical data on the actual status of medical care and results of specific health checkups in Japan as NDB Open Data for the public [9]. The NDB Open Data are suitable for a broad and plain understanding of Japanese healthcare [9]. This study aimed clarify the relationship between antihypertensive drug use and the number of people with high blood pressure based on the NDB Open Data.

## Methods

### Data source

The numbers of drug prescriptions by sex, age class, and prefecture were obtained from the NDB Open Data for FY 2018 [10, 11]. The NDB Open Data contain the top 100 drugs with the highest prescription numbers in each efficacy category. Additionally, the actual prescription quantities for products with fewer than 1000 prescriptions are anonymized [12]. These data include only the commodity names of drugs and do not contain information about drug ingredients. In this study, ingredient names were added by linking the National Health Insurance (NHI) Drug Price List published by the MHLW [13] with NHI drug codes in the NDB Open Data. In the NDB Open Data, laboratory values for every 5 years of age and by prefecture for those aged 40–74 years who underwent specific health checkups are available. The number of persons in each age group from 40 to 74 years with systolic and diastolic blood pressure in FY 2018 [11] was obtained.

### Study design

In Japan, 99.1% of hypertension treatments are performed in the outpatient department [14]. As such, the target antihypertensive drugs were those prescribed as outpatient (in-hospital or out-of-hospital) oral medications in efficacy categories 214 (antihypertensive drugs) and 217 (vasodilators). The WHO defines the Anatomical Therapeutic Chemical (ATC) classification, which classifies drugs into five levels. In this study, antihypertensive drugs were classified using the fourth level, ATC4 [15], which classifies drugs anatomy, therapy, pharmacology, and chemistry. The ATC4 and DDD of the target antihypertensive drugs used are shown in Table 1. DDD (g) was obtained using the ATC/DDD Index 2023 [16]. The DDDs of azelnidipine, efonidipine, and benidipine hydrochloride are not listed in the ATC/DDD Index 2023; therefore, they are defined as the maximum dose in the Japanese package inserts for these medications.

**Table 1:** ATC4, generic name, and DDD of antihypertensive drugs

Mechanism of action	ATC4^*1^	Generic name	DDD^*2^ (g)
Phosphodiesterase inhibitors	B01AC	Dipyridamole	0.400
Organic nitrates	C01DA	Isosorbide mononitrate Isosorbide dinitrate	0.040 0.060
Other vasodilators used in cardiac diseases	C01DX	Dilazep Nicorandil	0.100 0.040
Methyldopa	C02AB	Methyldopa	1.000
Alpha-adrenoreceptor antagonists	C02CA	Doxazosin Urapidil	0.004 0.120
Sulfonamides, plain	C03BA	Indapamide	0.003
Aldosterone antagonists	C03DA	Eplerenone	0.050
Beta-blocking agents, selective	C07AB	Metoprolol	0.150
Alpha- and beta-blocking agents	C07AG	Carvedilol	0.038
Dihydropyridine derivatives	C08CA	Amlodipine Azelnidipine Benidipine Cilnidipine Efonidipine Nifedipine	0.005 0.016^*3^ 0.004^*3^ 0.010 0.040^*3^ 0.030
Phenylalkylamine derivatives	C08DA	Verapamil	0.240
Benzothiazepine derivatives	C08DB	Diltiazem	0.240
ACE^*4^ inhibitors, plain	C09AA	Enalapril Imidapril	0.010 0.010
ARBs^*5^, plain	C09CA	Azilsartan Candesartan Irbesartan Losartan Olmesartan medoxomil Telmisartan Valsartan	0.040 0.008 0.150 0.050 0.020 0.040 0.080
ARBs^*5^ and diuretics	C09DA	Candesartan and diuretics Losartan and diuretics Telmisartan and diuretics Valsartan and diuretics	1 UD ^*6^
ARBs^*5^ and calcium channel blockers	C09DB	Azilsartan and Amlodipine Candesartan and Amlodipine Irbesartan and Amlodipine Olmesartan medoxomil and Azelnidipine Telmisartan and Amlodipine Valsartan and Amlodipine Valsartan and Cilnidipine	

^*1^ *ATC* Anatomical Therapeutic Chemical, ^*2^ *DDD* Defined daily dose, ^*3^ DDD is not listed in the ATC/DDD Index 2023 and is defined from the dosage and administration in the package insert. ^*4^ *ACE* angiotensin-converting enzyme inhibitor, ^*5^ *ARB* angiotensin II receptor blocker, ^*6^ *UD* Unit of combination drug

### Calculation of antihypertensive drug use

Antihypertensive drug use was evaluated using the DID. The number of prescriptions of antihypertensive drugs obtained from the NDB Open Data was converted to a titer, and the DID was calculated using the DDD and the population by sex and age class [17] or prefecture [18] in FY 2018, as reported by the Statistics Bureau of Japan, by using the following formula (1):

1 $$\textrm{DID}\ \left(\textrm{DDDs}/\textrm{1,000}\ \textrm{inhabitants}/\textrm{day}\right)=\textrm{Annual}\ \textrm{consumption}\ \left(\textrm{g}\right)/\textrm{DDD}\ \left(\textrm{g}\right)/\left(\textrm{population}/\textrm{1,000}\ \textrm{inhabitants}\right)/365\ \left(\textrm{days}\right)$$

### Calculation of the number of people with high blood pressure

The number of people with high blood pressure was calculated using formulas (2) and (3) for men and women aged 40–74 years.

2 $$\textrm{Number}\ \textrm{of}\ \textrm{people}\ \textrm{with}\ \textrm{high}\ \textrm{systolic}\ \textrm{blood}\ \textrm{pressure}\ \left(/\textrm{1,000}\ \textrm{inhabitants}\right)=\left(\textrm{number}\ \textrm{of}\ \textrm{persons}\ \textrm{with}\ \textrm{systolic}\ \textrm{blood}\ \textrm{pressure}\ \textrm{of}\ 140\ \textrm{mmHg}\ \textrm{or}\ \textrm{high}\textrm{er}/\textrm{Number}\ \textrm{of}\ \textrm{people}\ \textrm{whose}\ \textrm{systolic}\ \textrm{blood}\ \textrm{pressure}\ \textrm{had}\ \textrm{been}\ \textrm{registered}\right)\times \textrm{1,000}$$

3 $$\textrm{Number}\ \textrm{of}\ \textrm{people}\ \textrm{with}\ \textrm{high}\ \textrm{diastolic}\ \textrm{blood}\ \textrm{pressure}\ \left(/\textrm{1,000}\ \textrm{inhabitants}\right)=\left(\textrm{number}\ \textrm{of}\ \textrm{persons}\ \textrm{with}\ \textrm{diastolic}\ \textrm{blood}\ \textrm{pressure}\ \textrm{of}\ 90\ \textrm{mmHg}\ \textrm{or}\ \textrm{high}\textrm{er}/\textrm{Number}\ \textrm{of}\ \textrm{people}\ \textrm{whose}\ \textrm{diastolic}\ \textrm{blood}\ \textrm{pressure}\ \textrm{had}\ \textrm{been}\ \textrm{registered}\right)\times \textrm{1,000}$$

### Statistical analysis

The correlation between the DID of antihypertensive drugs and the number of people with high blood pressure according to sex and age class was evaluated using Spearman’s rank correlation coefficient. Each prefecture was divided into three regions (East, Central, and West), and differences in DID in each category were evaluated using the Kruskal–Wallis test and Bonferroni adjustment. The two-sided significance level was set at 5%, and Easy R (EZR) [19] was used for the analysis.

### Ethical consideration

This study was conducted in strict compliance with the “Ethical Guidelines for Medical Research Involving Human Subjects” and was approved by the Ethics Committee of Kyoto Pharmaceutical University (approval number: E21–011).

## Results

### The use of antihypertensive drugs by sex and age class in FY 2018

**Fig. 1:**
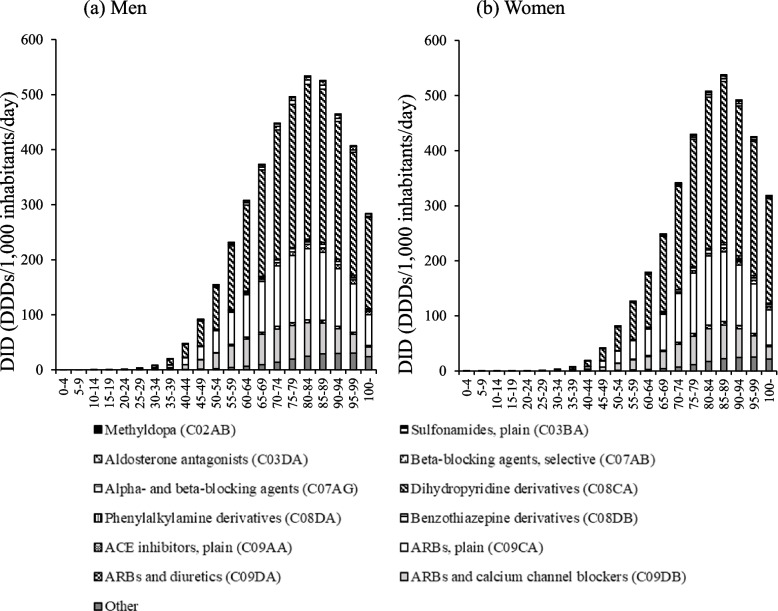
The trend for antihypertensive drug use stratified by sex and age class. Other: phosphodiesterase inhibitors (B01AC), organic nitrates (C01DA), other vasodilators used in cardiac diseases (C01DX), and alpha-adrenoreceptor antagonists (C02CA). DDD: Defined daily dose

The DID for antihypertensive drugs according to sex and age class in FY 2018 is shown in Fig. 1. DID increased with age for both men and women but decreased after peaking at ages 80–84 for men and 85–89 for women. Antihypertensive drug use was higher among men in the age group of 5–84 years and among women in the age group of ≥85 and older age groups.

**Fig. 2:**
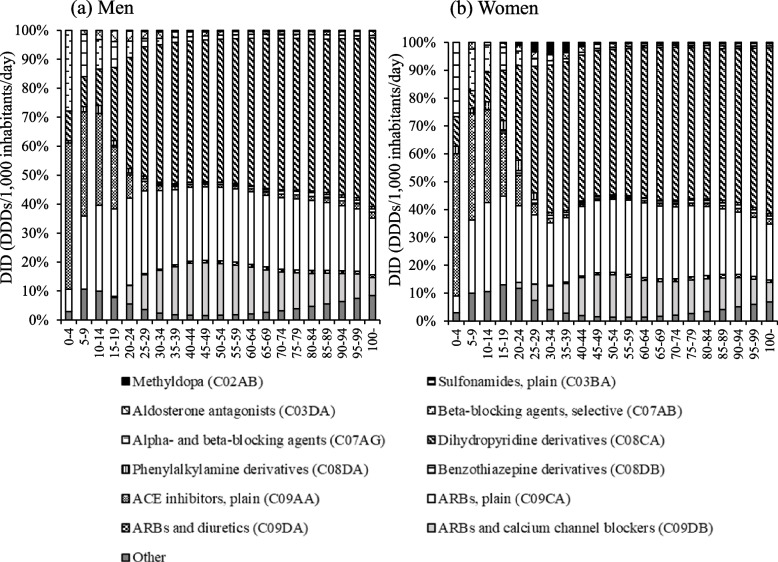
The proportion of antihypertensive drug use stratified by sex and age class. Other: phosphodiesterase inhibitors (B01AC), organic nitrates (C01DA), other vasodilators used in cardiac diseases (C01DX), and alpha-adrenoreceptor antagonists (C02CA). DDD: Defined daily dose

The proportions of DID for antihypertensive drugs according to sex and age class in FY 2018 is shown in Fig. 2. In both men and women, dihydropyridine (DHP) derivative Ca antagonists (C08CA and C09DB) and ARBs (C09CA, C09DA, and C09DB) were the main drugs used age group of≥20 years. On the other hand, the usage rates of ACE inhibitors, plain (C09AA) and alpha- and beta-blocking agents (C07AG), and others were higher among those aged 0–19 years than among those aged 20 years and higher. Enalapril accounted for more than 90.9% of the ACE inhibitor, plain (C09AA), use in both sexes in the age group of 0–19 years. In addition, alpha- and beta-blocking agents (C07AG) in the age group of 0–19 years included only carvedilol for both sexes.

Among women in their 20s–40s, methyldopa (C02AB) use ranged from 0.2–4.1%, compared with 0.01–0.19% among men in the same age group. The use of ARBs (C09CA, C09DA, and C09DB) ranged from 29.6–41.8%, which was lower than that in men of all ages.

### Relationship between the use of antihypertensive drugs and the number of people with high blood pressure

**Fig. 3:**
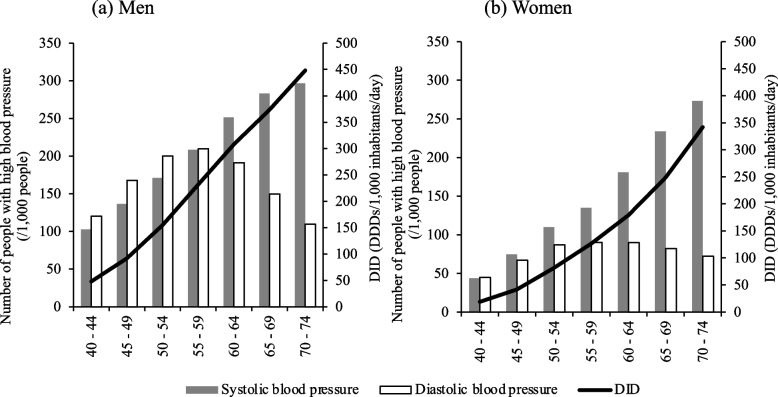
Relationship between antihypertensive drug use and number of people with high blood pressure. DDD: Defined daily dose The x-axis represents age in increments of 5 years

The relationship between DID and the number of people with high blood pressure by sex and age in the 40–74 years age group in FY 2018 is shown in Fig. 3. A very strong positive correlation was found between the number of people with high systolic blood pressure and the DID in both sexes (men: *r* = 1, *P* < 0.05; women: *r* = 1, *P* < 0.05). In contrast, there was no significant correlation between the number of people with high diastolic blood pressure and DID in either sex (men: *r* = − 0.214, *P* > 0.05; women: *r* = 0.393, *P* > 0.05).

### Antihypertensive drug use by prefecture in FY 2018

**Fig. 4:**
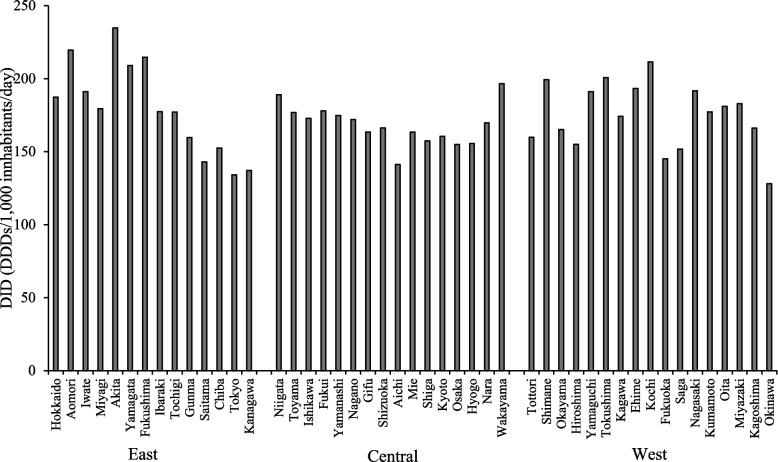
The trend for antihypertensive drug use stratified by prefecture. DDD: Defined daily dose

Figure 4 shows the DID for antihypertensive drugs in each prefecture in FY 2018. The prefecture with the highest DID was Akita (234.7) and that with the lowest DID was Okinawa (128.1). There was a large difference in the DID for each prefecture. The median (range) DID was 178.4 (134.1–234.7), 168.0 (141.2–196.6), and 177.3 (128.1–211.5) in the east, central, and west regions, respectively. No significant differences were found among the regions in terms of DID (*P* > 0.05).

## Discussion

In this study, the use of antihypertensive drugs in Japan, based on the NDB Open Data, was clarified for the first time using DID. Furthermore, there was a very strong positive correlation between the number of people with high systolic blood pressure and the DID among men and women aged 40–74 years. In addition, this study revealed, for the first time, the use of antihypertensive drugs in each prefecture of Japan. The methodology used in this study may be useful for the continued assessment of antihypertensive drug use and changes in the number of people with high systolic blood pressure based on the changes in antihypertensive drug use.

Antihypertensive drug use increased with age in both men and women (Fig. 1). This is thought to be partly because hypertension is more likely to develop with age owing to a decrease in vascular elasticity and baroreceptor reflex impairment [2]. Furthermore, a very strong positive correlation was found between the number of people with high systolic blood pressure and DID in both men and women aged 40–74 years (Fig. 3). In contrast, there was no significant correlation between the number of people with high diastolic blood pressure and DID (Fig. 3). It has been reported that progression of atherosclerosis leads to an increase in systolic blood pressure and a decrease in diastolic blood pressure, with the prevalence of isolated systolic blood pressure increasing and that of isolated diastolic hypertension decreasing with advancing age [2, 20]. Therefore, it is possible that the progression of arteriosclerosis associated with advanced age may be a contributing factor. In addition, the use of antihypertensive drugs was higher among men in the age group of 5–84 years and among women in the age group of 85 years and higher (Fig. 1). In the 30–79 years age group hypertension prevalence in men has been reported to be higher than that in women [21]. In addition, women have a longer average life expectancy [22], which may have contributed to this result.

In both men and women, DHP derivative Ca blockers (C08CA and C09DB) and ARBs (C09CA, C09DA, and C09DB) were the main drugs used from the age of 20 years (Fig. 2). These results are similar to those of a previous study using Diagnostic Procedure Combination (DPC) data [23]. Although the DPC data can only capture the use of drugs in some hospitals, the use of NDB Open Data has made it possible to capture the use of drugs nationwide. Guidelines for the management of hypertension in Japan [2] include Ca channel blockers, ARBs, ACE inhibitors, and diuretics as first-line drugs for hypertension management. Among these, DHP derivatives and Ca channel blockers have been reported to have strong hypotensive actions and have been indicated for many patients in Japan [2]. In addition, ARBs have organ-protective effects [24, 25], and the low frequency of adverse effects and high tolerability [26] associated with them are thought to contribute to their widespread use. However, the use of antihypertensive drugs varies across countries [5]. ACE inhibitors are mainly used in foreign countries [27, 28] but not so much in Japan. Dry cough, a side effect of ACE inhibitors, has been shown to develop more frequently in East Asians than in Caucasians [29]. Therefore, the use of ACE inhibitors is considered low in Japan.

Both men and women aged 0–19 years had higher rates of the use of ACE inhibitors, plain (C09AA), alpha- and beta-blocking agents (C07AG), and other drugs than those in their 20s and older (Fig. 2). Enalapril accounted for more than 90.9% of the use of ACE inhibitors plain (C09AA) in both sexes in the age group of 0–19 years (Fig. 2). Enalapril can be administered as early as 1 month of age. Therefore, enalapril is likely used more frequently than other antihypertensive drugs. Furthermore, carvedilol was the only alpha- and beta-blocking agent (C07AG) used in the age group of 0–19 years. In addition to hypertension, carvedilol is indicated for rapid atrial fibrillation, angina pectoris, and chronic heart failure due to ischemic heart disease or dilated cardiomyopathy. Because this study used ATC codes to classify antihypertensive drugs, it was not possible to evaluate the intended use of antihypertensive drugs with multiple indications, such as carvedilol. In the future, it will be necessary to establish a system for evaluating drug use.

A higher percentage of women in their 20s–40s used methyldopa (C02AB) than their mens’ counterparts (Fig. 2). Methyldopa is used more frequently in Japan because it is considered one of the first choices for the treatment of gestational hypertension in the Japanese guidelines for the management of hypertension [2]. In addition, ARBs are contraindicated in pregnant women [2] because of fetal and neonatal deaths and malformations that occur in patients who receive ARBs in the second and third trimesters of pregnancy [30, 31]. In this study, the rate of ARB (C09CA, C09DA, and C09DB) use was lower among women in their 20s–40s than in their mens’ counterparts (Fig. 2).

In this study, a large difference was observed in the use of antihypertensive drugs in each prefecture (Fig. 4), which was determined for the first time. In 2018, the Japanese Society of Hypertension set the goal of reducing the number of patients with hypertension by 7 million over 10 years and extending healthy life expectancy [32]. Previous reports have shown that the trend in drug use in more narrow regions such as municipalities differs from the national and prefectural usage trends [33]. Therefore, it is necessary to understand the trends in antihypertensive drug use in each region and implement appropriate countermeasures [33]. The methodology used in this study can be used to assess changes in the number of people with high systolic blood pressure based on the changes in antihypertensive drug use in a specific region. Therefore, targeted management is considered useful to reduce the number of patients with hypertension.

This study had several limitations. First, information on diagnoses was not available, and drugs prescribed for conditions other than hypertension may have been included in the study. Second, because NDB Open Data were used, factors not included in the data, such as public assistance, could not be considered. In addition, fewer than 1000 prescriptions were anonymized, which may have led to an underestimation of the use of antihypertensive drugs in Japan. Third, the number of people with high blood pressure calculated in this study was based on the results of the specific health checkups and may differ from the actual number of people with high blood pressure. Despite these limitations, this study is useful in understanding the use of antihypertensive drugs in Japan.

## Conclusion

In this study, the use of antihypertensive drugs in Japan was clarified for the first time according to sex, age, and prefecture based on the ATC classification. In addition, our findings suggest that the DID of antihypertensive drugs can be used as an alternative indicator of the number of people with high systolic blood pressure. In the future, the use of antihypertensive drugs in each prefecture will be evaluated over time, making it possible to easily assess the efforts undertaken to reduce the number of people with high blood pressure in each region.

## Data Availability

The NDB Open Data used in this study are publicly available and can be obtained from the following URL: https://www.mhlw.go.jp/stf/seisakunitsuite/bunya/0000177182.html.

## Abbreviations

ACE: Angiotensin-converting enzyme
ARB: Angiotensin II receptor blocker
ATC: Anatomical Therapeutic Chemical
Ca: Calcium
DDD: Defined daily dose
DHP: Dihydropyridine
DID: DDDs/1000 inhabitants/day
DPC: Diagnostic Procedure Combination
MHLW: Ministry of Health, Labour and Welfare
NDB: National Database of Health Insurance Claims and Specific Health Checkups of Japan
NHI: National Health Insurance
OECD: Organization for Economic Co-operation and Development
WHO: World Health Organization
